# The research performance of Iranian medical academics: a National Analyses

**DOI:** 10.1186/s12909-019-1892-4

**Published:** 2019-12-03

**Authors:** Homayoun Sadeghi-Bazargani, Fahimeh Bakhtiary, Mina Golestani, Yasin Sadeghi-Bazargani, Nazila Jalilzadeh, Mohammad Saadati

**Affiliations:** 1 Evidence based Medicine Research Center, Tabriz University of Medical Sciences, Tabriz, Iran; 2 Road Traffic Injury Research Center, Health Management and Safety Promotion Research Institute, Tabriz University of Medical Sciences, Tabriz, Iran; 3 Medical student, student research committee, Tabriz University of Medical Sciences, Tabriz, Iran; 4 Drug Applied Research Center, Tabriz University of Medical Sciences, Tabriz, Iran; 5 Road Traffic Injury Research Center, Tabriz University of Medical Sciences, Tabriz, Iran

**Keywords:** Scientometry, Medical research, Research performance, Evaluation

## Abstract

**Background:**

Scientometric studies are one of the most important and useful tools to assess the research performance and knowledge impact of researchers. The aim of this study was to map out the scientific performance of the Iranian medical academics with respect to a detailed range of scientometric indicators.

**Methods:**

Using scientometric approach, individual and scientific performance data of medical academic staff were extracted from the Iranian Scientometric Information Database (ISID). Total number of publications, total number of citations, citation per paper, h-index, international collaboration, self-citation, SJR decile, i10-Index, Quartile distribution were the studied scientometric variables. Out of the registered 19,023 academic staff, 746 were included in the study through simple random sampling method using random sample extraction function in STATA. Data were analyzed using STATA 14 statistical software package.

**Results:**

Most of the included academicians were men (60%). A total of 13,682 articles were published by them until 2018, being cited 114,928 times with a mean of 5.77 citation per paper. H-index median was three and about 90% of the staff had an H-index below 10. Number of published papers, cite per paper and H-index metrics were significantly different with respect to gender, academic position/degree, and general field of study (*p* < 0.05). About 2.5% of published articles were contributed through international collaboration. The scientometric performance of academic staff was highly diverse with respect to the employing institution and its national classification group (type 1, 2, 3).

**Conclusions:**

Nevertheless to the great scientific production of medical academics, individual and institutional characteristics were identified as effective variables in academics research performance and should be considered in their assessment. Academicians affiliated with type 2 and 3 universities (based on national ranking of medical universities) had weaker research performance compared to those affiliated with type 1 universities. However, low rate of international research collaborations was a common challenge in medical universities.

## Background

Science and technology progress is a vital base for sustainable social development of the countries in the world. Allocating resources such as human resource, infrastructure and budget for research is considered a high priority in both developed and developing countries [1]. Creation of international research collaboration networks and teams shows the global importance of science advancement worldwide [2, 3]. Literature revealed that developed countries spend on average 1.5% of their gross domestic product (GDP) versus a figure of about 0.5% in Iran on science and technology [4].

One of the most important policies in Iran, after the Islamic revolution, was promoting scientific growth. The urgent need for developing an effective health system emphasizes this importance, because providing high quality health services depends on production, publication, dissemination and use of updated knowledge to promote population health status. Therefore, medical research came into attention and many researchers started working on biomedical issues [5].

Evidence supports Iran’s impressive growth in science production such that Butler (2006) reported Iran to hold the second rank after Turkey among the Islamic countries, with respect to the number of published articles [6]. Moreover, Malekzadeh et al., revealed that the number of articles by Iranian researchers indexed in Web of Science Core Collection, has experienced an increase of 26 folds during 2000 to 2014 [7]. Moreover, it was reported by previous studies that Iran has 0.29% relative share of the global scientific output in 2003 versus a figure of 0.0003% in 1970 [8]. Analyzing scientific performance of institutions, universities and researchers has become an inevitable and important priority [9]. The result of scientometric analysis can be used for policy-making on research budgeting and promotion. Moreover, the results could prove useful for ranking universities and institutions [10–12]. The research performance of universities are measured and analyzed using scientometric indicators in Iran [13]. Regarding the growth of research in Iran, scientometric indicators such as “number of publications”,” citations”, “H-index”, etc. has been used to analyze scientific activities. Previous studies comparing the universities of medical sciences in north and west of Iran, based on their research performance, revealed that despite the growth in scientific publications, the incremental trend has not been stable [14, 15]. A 5-year analysis of scientific performance of research centers affiliated with Tehran University of Medical Sciences showed that, overall, 4867 papers were published and the median of paper publication per author was 2.8 [16].

Despite the previous literature on scientific performance of the Iranian academic staff, which were mostly focused on scientometrics of institutes/ universities using summarized institutional indicators, studies using data at individual level have been mostly local. It seems that providing a comprehensive analysis of scientometric indicators regarding institutional and individual characteristics such as academic position and field of study was investigated. However, collaborated scientific production was ignored in most previous studies. Moreover, performing a country-wide analysis with adequate generalizibity seemed to be necessary in order to provide medical education policy-makers as a foundation to make future medical research policies in Iran. The aim of this study was to map out the scientific performance of Iranian medical academics with respect to a detailed range of scientometric indicators.

## Methods

In a cross-sectional study, the scientific performance of Iranian medical academic staff was investigated in 2018 through a scientometric approach. All the academic staff working either as permanent or time-restricted employment in all Medical universities throughout the country were enrolled into the study. No restriction was applied with respect to field of education, academic degree or academic position of the participants.

### Sampling

A total of 19,023 faculty members were identified from Iranian Scientometric Information Database - ISID. A sample of 746 persons (Using n = (Z^2^*SD^2^)/d^2^ where SD = 4.74 and d = 0.25) were selected for detailed assessment of their recorded scientific performance. The selection process was conducted using simple random sampling method by means of the random sample extraction function in STATA statistical software package.

### Study setting

ISID is a national scientometric information database for Iranian Medical Universities [17]. The ISID provides research performance data for medical universities academicians based on Scopus database. Academic members’ scientific performance information extract and update, regularly.

### Variables

The scienttometric information as well as background characteristics of the participants was extracted from the data deposited in ISID. Key variables included background information comprising academic staff gender, academic position, academic degree, general field of study and universities ranking (1, 2, 3) and being in metropolis and Scientometric data as following (Table 1):

**Table 1:** Scientometric variables in the study

N	Name	Definition
1	Total Number of publications	Total number of published Scopus-indexed articles by individuals
2	Total Number of citations	Total number of citations to published literature by individuals
3	Citation per paper	Is calculated by dividing the total number of citations by the total number of published papers
4	H-index	Is defined by how many h of a researcher’s publications each have at least h citations
5	International collaboration	Shows the number of publications in collaboration with researchers from countries other than Iran
6	Self-citation	Shows the share of citations done by the researchers themselves
7	SJR 10% (decile)	The number of articles which were published in 10% top journals based on SJR ranking
8	i10-Index	The number of articles with at least 10 citation based on Scopus
9	Quartile distribution	The number of published articles in journals in each quartile based on journal Impact Factor, CiteScore, SJR and SNIP

Individuals research work history was calculated using the formula ((2018 – Year of First Scopus-indexed article publishing year) + 1) using ISID data.

### Data extraction and analysis

Data were extracted by two of the researchers (FB and NJ) using a goal-driven researcher-made data collection form. To ensure intra-rater agreement, 30 records were randomly selected and the data extraction and classifications were done independently by the two data extractors and their agreement was assessed and confirmed. Descriptive statistics were used to describe the scientometric indicators including frequency distribution, mean, median and standard deviation (SD). Shapiro-Wilk W test showed that data does not have normal distribution. Regarding, Mann-Whitney and kruskal-wallis non-parametric tests were used to study the relation between background characteristics and scientometric indicators. To adjust the research work history on the selected scientoemtric indicators, the 90% percentile for indicators were used for categorizing and logistic regression was used. The binary logistic regression models outcomes were having H-index, Citation per paper and Number of published papers above 90% percentile in each indicator. STATA version 14 software package was used for data analysis.

## Results

About 60% of the participants were men. Only 13.2% of the participants held a Professor position and 52.7% were assistant professors. Most of the universities (72.6%) did not have any academic staff enrolled with a Professor position. Most of the participants (38%) held PhD degrees and mostly were from Iran Medical University, Isfahan Medical University and Ahvaz Medical University, respectively. A total of 13,682 papers were published by the participants until May 2018. The median for number of published papers was 9.5 (min = 1, max = 227). The articles were cited 114,928 times with an average citation per paper of 5.77 varying from zero to 173.7. The majority of the subjects (60.1%) had less than five citations per paper. Only 13.5% of the subjects had a citation per paper index above 10. H-index median was 3 ranging from zero to 40. Nearly 90% of the staff had an H-index metric below 10. Moreover, the median for i10-Index was 2 (ranging from zero to 41).

Results revealed a significant difference between men and women in H-index (*p* < 0.02) and Number of published papers (*p* < 0.001) metrics. Scietometric status according to academician’s individual characteristics and adjusted result by research work history are presented in Table 2.

**Table 2:** Scietometric indexes status regarding academician’s individual variables

Individual predictors	N	H-index			Citation per paper			Number of published papers		
		Median (iqr)	Sig.	Sig1*	Median (iqr)	Sig.	Sig1*	Median (iqr)	Sig.	Sig1*
Gender										
Male	452	3 (4)	< 0.02	0.55	4.42 (5.4)	0.04	0.65	11 (17.5)	< 0.001	0.11
Female	292	3 (4)			3.6 (4.72)			8 (12.5)		
Academic position										
Instructor	80	2 (2)	< 0.001	0.004	3.08 (3.9)	< 0.001	0.33	4 (4)	< 0.001	0.009
Assistant Professor	392	2 (3)			3.31 (4.3)			7 (9)		
Associate Professor	174	5 (4)			4.6 (4.5)			15.5 (19)		
Professor	98	9 (7)			7.11 (5.64)			32.5 (46)		
General field of study										
Public health	119	3 (4)	< 0.001	0.008	3.66 (4.2)	< 0.001	0.47	10 (21)	< 0.001	< 0.001
Nursing	41	2 (2)			2.5 (2.75)			5 (6)		
Para-medical	39	3 (2)			4 (4.12)			6 (7)		
Medicine	407	4 (4)			4.05 (4.9)			11 (16)		
Dentistry	48	2 (2)			3.16 (3.54)			4 (6)		
Pharmacy	56	7 (8.5)			7.88 (6.78)			19.5 (40.5)		
Other	20	5.5 (4.5)			5.75 (5.89)			15.5 (39)		
Un-related to medical field	14	3.5 (3)			3.23 (5.06)			10.5 (10)		
Academic degree										
Public Health PhD	283	4 (6)	< 0.001	0.3	4.84 (5.58)	< 0.001	0.93	13 (23)	< 0.001	0.3
PhD- Pharmacy	30	7.5 (10)			9.08 (6.61)			27.5 (39)		
MD Specialty Degree	192	3 (3)			3.67 (4.93)			9 (12)		
PhD-Dental	46	2 (2)			3 (3.33)			4 (6)		
MD (GP)	4	4.5 (5.5)			5.21 (4.21)			17 (22.5)		
Medical Postdoc	98	3.5 (5)			3.7 (4.44)			8.5 (15)		
Fellow	19	4 (3)			4.6 (6.5)			16 (16)		
MSc	72	1.5 (2)			3 (3.55)			4 (4)		

*Adjusted *p*-value by research work history-binary logistic regression

More than 49% of the staff did not have any publication with international collaboration. Only 2.5% (*n* = 343) of the published articles had international contribution. International collaboration in scientific publication was significantly higher in academicians affiliated with universities in metropolises versus other cities (*p* < 0.001) and similarly in men versus women (*p* < 0.002). Moreover, the distribution of international collaboration rate was found to be significantly different among various academic degrees (*p* < 0.0001). Moreover, metropolises universities and staff affiliated with type 1 universities, had the highest percentage of publications with international collaboration. Academics affiliated with Baqiyatallah University of Medical Sciences and Pasteur institute of Iran had the highest number of publications with international collaboration.

More than 61% of the academic staff had no publication in top 10% of journals according to the SJR ranking. About 57% of them had no publication in Q1 journals (highest Impact Factor quartile) and only 6.4% had more than 20 publications in these journals. Regarding Cite Score, SJR and SNIP metrics, respectively, 32.34, 32.66 and 27.04% of the Iranian medical academics had no publications in Q1 journals (highest Cite Score, SJR and SNIP quartiles).

Academic staff affiliated with Pasteur institute, Tehran and Zahedan University of Medical Sciences had the highest mean of H-index, respectively. K-wallis test showed that number of published papers was significantly different among the three ranking types of universities (*p* < 0.001). Scietometric status in Iranian Medical Universities are presented in Table 3.

**Table 3:** Scietometric indexes status in Iranian Medical Universities (Top 10 Universities**)

N	University	Staff*(N)	H-index		Sjr 10%		Cite per paper	
			Mean (SD)	Median (iqr)	Mean (SD)	Median (iqr)	Mean (SD)	Median (iqr)
1	Tehran	84	7.09 ± 7.50	4(6.5)	4.88 ± 6.13	2(8)	6.5 ± 5.78	5(6.26)
2	Tarbiat Modares	5	4 ± 1.22	4 (1)	6 ± 7	2(8)	6.40 ± 4.30	5.09(1.91)
3	Shahid Beheshti	65	5.38 ± 3.99	4(5)	3.98 ± 4.80	1(8)	8.59 ± 21.3	5(4.9)
4	Mashhad	40	3.85 ± 3.02	3(2)	3.82 ± 4.31	1(8)	4.45 ± 3.39	3.68(2.44)
5	Shiraz	31	6.38 ± 5.04	5(7)	5.65 ± 6.81	2(10)	5.74 ± 3.55	5.33(6.05)
6	Isfahan	34	4.55 ± 2.56	4(3)	3.81 ± 4.6	1 (4.5)	5.16 ± 3.98	3.79 (2.6)
7	Tabriz	41	4.75 ± 3.59	4(5)	2.87 ± 3.63	1(1)	5.35 ± 4.15	4.33(4.45)
8	Iran	36	4.69 ± 4.71	3(3)	3.5 ± 5.45	1(1)	5.75 ± 6.71	3.73(5)
9	Kerman	20	4.05 ± 2.64	3.5(3.5)	3.05 ± 4.32	1(1)	5.63 ± 4.9	3.60(6.34)
10	Ahvaz	27	2.81 ± 2.64	2(2)	1.61 ± 2.35	1(0)	3.96 ± 3.51	2.75(3.04)

*Number of staff from each university included in the study

**Based on webometrics report, 2018

## Discussion

Results revealed diversity in scientific performance of medical academics affected by institutional and individual characteristics. General field of study was one of the factors which explicitly affected the scientific performance of the staff. Academics in Pharmacy field had remarkably higher median in number of published articles, citations per paper, and H-index. Eskrootchi and et al. (2007) in a study on Iranian Medical scientific papers during 1978–2007, reported that papers published in pharmacy field had the highest growth. In their study period, 2222 papers with 10,976 citations were published in pharmacy field by Iranian medical researchers [18]. In a study on Iranian medical publications indexed/archived in PubMed till 2015, pharmacological and phytochemical studies had the highest share (14.7%) [19]. This reveals the reality of research performance differences among academics with various fields of study. Subject-adjusted research performance evaluation and quality assessment has been proposed in previous studies [20, 21]. Some fields, such as pharmacy, have a vast capacity for research through laboratories. While other fields, such as public health, have more difficulty and need longer time to conduct a research and publish the results as they work with social structures and mostly with human populations with its complexities. Therefore, assessing the academics research performance should be adjusted considering field characteristics using modeling studies based on available data. This may provide an adjusted tool for scientific performance assessment with respect to individual and institutional characteristics.

It was revealed that type 1 Medical universities in Iranian metropolises such as Tehran, Tabriz and Shiraz have significantly higher scientific outcomes than other medical universities in Iran. Moreover, these universities have the highest share of staff with higher academic positions. Results of a study by Rasoulabadi et al. (2015) also indicated that the trend of scientific publications in Iranian medical universities although increasing, has not been stable [14]. Research policies in Iran might be developed in an equitable way to encourage and promote scientific production in small universities. It was evidenced in a previous study in Shahid Beheshti Medical University, that employing new research policies comprising providing research infrastructures and re-engineering the research administrative processes, had led to increase in scientific production during 2009 to 2011 [22]. Developing new decentralized research agenda, taking into account the infrastructure, resources and capabilities of various universities, using local potentials such as organizing research donors, will promote the research activities equally across the country. Moreover, employing proper strategies for research capacity building in type 2 and type 3 universities could be useful.

International collaboration in research is an increasingly common pattern and essential for many areas of science [2]. Research collaboration not only benefits the researchers but also it is an advantage for organizations and also increases the research quality [23, 24]. It has been declared that collaborative work will lead to higher scientific impact because of multidisciplinary research team sharing different skills and abilities [23, 25]. It was revealed that only 2.5% of published articles were produced through international collaboration. Moreover, results revealed a smaller share of researchers from type 2 and type 3 medical universities and female researchers in international research collaborations. As collaboration brings synergy of researchers’ skills and expertise, costs and resources, technologies and knowledge sharing, it is necessary to encourage the collaboration, especially in type 2 and type 3 universities in Iran. This will lead to higher science impact of Iranian researchers and offers insight to more important issues concerning national and international health. Furthermore, beside the international collaboration, collaboration between medical universities should also be encouraged to have a monolith promotion of the universities and researchers.

Moreover, it should not be ignored that part of the Iranian research materials, and also other countries with non-English language, are not covered by indexing databases due to the so called language bias [14, 26]. The vast majority of international databases that evaluate scientific products only or adequately archive English articles. Scopus accepts English articles and non-English articles that have English abstract. Therefore, Iranian universities are required to apply policies for strengthening English knowledge, especially in the academic writing, so that they can showcase their production and increase their perspective and promote their academic rank among other universities. Providing educational workshops and other sources of information for university researchers, it has a great impact on the academic performance of universities.

As a strength, this study investigated the individual and institutional characteristics potentially affecting the research performance based on ISID database. One limitation with current study was the restriction of performance analyses only to the Scopus database.

## Conclusions

Iranian Medical Academic staff have made great effort conducting research in various fields. The academics research performance varied highly over individual and institutional characteristics. Academicians affiliated with low ranked universities (type 2&3) had weak research performance compared to type 1 universities. However, low rate of international research collaborations was a national challenge observed in all medical universities.

## Data Availability

Data will be available in case of request from the corresponding author.

## Abbreviations

GDP: Gross Domestic Product
ISID: Iranian Scientometric Information Database
SD: Standard Deviation
SJR: Scientific Journal Rankings
